# Methods for Evaluating the Combined Effects of Chemical and Nonchemical Exposures for Cumulative Environmental Health Risk Assessment

**DOI:** 10.3390/ijerph15122797

**Published:** 2018-12-10

**Authors:** Devon C. Payne-Sturges, Madeleine K. Scammell, Jonathan I. Levy, Deborah A. Cory-Slechta, Elaine Symanski, Jessie L. Carr Shmool, Robert Laumbach, Stephen Linder, Jane E. Clougherty

**Affiliations:** 1 Maryland Institute for Applied Environmental Health, University of Maryland School of Public Health, 2234 L SPH, 255 Valley Drive, College Park, MD 20742, USA; 2 Department of Environmental Health, Boston University School of Public Health, 715 Albany St., TW4, Boston, MA 02118, USA; mls@bu.edu (M.K.S.); jonlevy@bu.edu (J.I.L.); 3 Department of Environmental Medicine, University of Rochester Medical Center, 601 Elmwood Avenue, Box EHSC, Rochester, NY 14642, USA; deborah_cory-slechta@urmc.rochester.edu; 4 The Southwest Center for Occupational and Environmental Health, Department of Epidemiology, Human Genetics and Environmental Sciences, The University of Texas Health Science Center at Houston (UTHealth) School of Public Health, 1200 Pressler Street, Suite W-1030, Houston, TX 77030, USA; elaine.symanski@uth.tmc.edu; 5 Department of Environmental & Occupational Health, University of Pittsburgh, Bridgeside Point 1, 100 Technology Drive, Suite 350, Pittsburgh, PA 15219, USA; jessielcarr@gmail.com; 6 Environmental and Occupational Health Sciences Institute, Rutgers School of Public Health, 170 Frelinghuysen Rd, room 204, Piscataway, NJ 08854, USA; laumbach@eohsi.rutgers.edu; 7 Institute for Health Policy, School of Public Health, The University of Texas Health Science Center at Houston, 1200 Pressler, Houston, TX 77030, USA; stephen.h.linder@uth.tmc.edu; 8 Dornsife School of Public Health, Drexel University, 3215 Market Street, Nesbitt Hall, Office 616, Philadelphia, PA 19104, USA; jcloughe@drexel.edu

**Keywords:** cumulative risk, methods, community health, environmental justice, psychosocial stressors

## Abstract

Cumulative risk assessment (CRA) has been proposed as a means of evaluating possible additive and synergistic effects of multiple chemical, physical and social stressors on human health, with the goal of informing policy and decision-making, and protecting public health. Routine application of CRA to environmental regulatory and policy decision making, however, has been limited due to a perceived lack of appropriate quantitative approaches for assessing combined effects of chemical and nonchemical exposures. Seven research projects, which represented a variety of disciplines, including population health science, laboratory science, social sciences, geography, statistics and mathematics, were funded by the US Environmental Protection Agency (EPA) to help address this knowledge gap. We synthesize key insights from these unique studies to determine the implications for CRA practice and priorities for further research. Our analyses of these seven projects demonstrate that the necessary analytical methods to support CRA are available but are ultimately context-dependent. These projects collectively provided advancements for CRA in the areas of community engagement, characterization of exposures to nonchemical stressors, and assessment of health effects associated with joint exposures to chemical and psychosocial stressors.

## 1. Introduction

Many low income and minority populations and communities face disproportionate burdens from multiple, often co-occurring, environmental hazards, in conjunction with elevated exposures to social inequities and psychosocial stressors (e.g., financial strain, housing instability, discrimination, community violence). There is growing evidence that nonchemical stressors, including but not limited to psychosocial stressors, heighten vulnerability to the adverse health effects of chemical exposures [1,2,3,4,5,6,7,8]. Therefore, accurate assessment of the human health effects of chemical exposures requires a better understanding of the combined effects of chemical and previously or co-occurring nonchemical exposures.

Cumulative risk assessment (CRA) has been proposed as a means of evaluating possible additive and synergistic effects of multiple chemical, physical and social stressors on human health, with the goal of informing policy and decision-making, and protecting public health. The US Environmental Protection Agency (EPA) defines CRA as an analysis, characterization and possible quantification of the combined risks to health or the environment from multiple agents or stressors consisting of three phases (1) planning, scoping and problem formulation; (2) analysis; and (3) interpretation and risk characterization [9]. While EPA has put forward several CRA framework guidance documents, stakeholders and independent advisory bodies (e.g., the National Academy of Sciences, the National Environmental Justice Advisory Council) continue to provide strategies for EPA to improve risk assessment and risk management practices to better account for multi-stressor exposures that cumulatively impact community and population health [10,11,12,13,14]. Specifically, the National Research Council’s Committee on Improving Risk Analysis Approaches used by EPA proposed that CRA be defined as evaluating an array of stressors (chemical and nonchemical) to characterize quantitatively—to the extent possible—human health or ecological effects, accounting for population vulnerability and background exposures [13]. The approach to CRA thus far tends to build upon the longstanding four-step framework for conducting single chemical risk assessment: hazard identification, dose-response assessment, exposure assessment, and risk characterization [15].

To support the development of CRA methods across the four steps of risk assessment, the EPA issued a research solicitation, Understanding the Role of Nonchemical Stressors and Developing Analytic Methods for Cumulative Risk Assessments, through its Science to Achieve Results (STAR) extramural research program in 2009. Although EPA has defined nonchemical stressors broadly to include physical and biological stressors such as noise, radiation, infectious agents, and poor nutrition [16], the research solicitation highlighted social conditions contributing to chronic psychosocial stress. Under this research solicitation, EPA awarded seven STAR CRA grants (see Table 1), with a total investment of $7 million in 2010, to support community-engaged research to develop new analytical tools for combining chemical and nonchemical stressors for community-based cumulative risk assessments and evaluating modification of health effects of chemical exposures by social stressors [17].

Results from these grants provide the “building blocks” for conducting CRAs to improve environmental health decision-making at national, state, and local levels. In the following section, we review the biological basis for incorporating psychosocial stressors in environmental health risk assessment, the motivation for the EPA STAR CRA program. Then we present the broad range of methods employed by the studies funded under the STAR CRA mechanism, which represent a variety of disciplines, including population health science, laboratory science, social sciences, geography, statistics and mathematics. Using various techniques, the studies examined combined effects and developed insight regarding how psychosocial stressors influence population susceptibility and vulnerability to chemical stressors. We conclude with a discussion of how the insights from these studies could inform CRA practice as defined by EPA, along with lessons learned and priorities for further research.

## 2. Psychosocial Stressors in Cumulative Risk Assessment

The prospect of assessing the effects of multiple stressors is daunting, as there are a seemingly an infinite number of multiplicative combinations of agents, exposures, targets, or health endpoints, with varying temporal patterns occurring acutely and/or chronically over the life span. Approaches to these many-to-many-relationships can be simplified using models in which multiple stressors affect the same physiological systems and/or share common mechanisms of action [18]. These more-practical models for CRA require some understanding of common biological pathways or targets [12,13]. Below, we briefly describe the mechanisms by which psychosocial stress can influence susceptibility, to help frame the research strategies developed by the various STAR grants.

Psychosocial stress occurs when an external stressor (an event or condition that is in some way demanding or threatening) overwhelms an individual’s perceived coping capacity and resources [19]. Acute and chronic stress impact health through a wide range of biological pathways, including the hypothalamic-pituitary-adrenal (HPA) and sympathetic-adrenal-medullary (SAM) axes, and other key regulatory systems of the body, including immune, endocrine, central nervous system, cardiovascular, respiratory, and metabolic functions [20,21,22].

In response to acute stress, the hypothalamus secretes corticotrophin releasing factor (CRF), which acts on the pituitary gland, causing release of adrenocorticotrophic hormone (ACTH), which subsequently acts on the adrenal gland, resulting in the release of cortisol (corticosterone in animals). Normally, cortisol, the primary human stress hormone, exhibits both ultradian and circadian rhythms, with low nocturnal concentrations that increase to a morning high controlled by a circadian clock [23]. Release of glucocorticoids is terminated by a classic negative feedback loop, whereby the unbound form inhibits any further adrenocorticotrophic hormone (ACTH) release from the pituitary gland. This inhibition is further facilitated by glucocorticoid binding to specific brain regions rich in glucocorticoid receptors, such as the hippocampus and other structures of the limbic system [24]. The HPA axis also has significant interactions with the brain mesocorticolimbic dopamine/glutamate system [25,26,27,28]. Acute stress also activates the SAM axis, resulting in immediate autonomic nervous system activation and release of catecholamines at sympathetic neuron synapses (norepinephrine) and from the adrenal medulla (epinephrine) into the circulatory system.

Importantly, the body’s response to stress is adaptive, meaning it responds to changing conditions and demands. However, over-extension of this acute stress response over time (a.k.a., chronic stress) [29], and the consequent failure to achieve homeostasis is what is thought to ultimately impose physiological damage and increase an individual’s susceptibility to injury from other social and environmental stressors [30,31]. This condition is commonly referred to as ‘allostatic load’, and includes systemic impacts to cardiovascular, immune, endocrine, and metabolic function [32], as well as neurodevelopmental and cognitive effects [33,34]. A contrasting model in which cortisol production is decreased following chronic stress has been proposed to explain the pro-inflammatory effects of chronic stress [35]. Chronic, repeated activation of acute stress responses may down-regulate, or blunt, adaptive acute stress responses either via dis-regulation of cortisol or catecholamine release, at the target tissue [30,36,37]. A meta-analysis found that chronic stress was associated with a dysregulated pattern of secretion of cortisol, including lower morning cortisol but higher secretion during the rest of the day, giving rise to a flattened diurnal variability [38].

Psychosocial stressors and chemical pollutants impact many of the same physiological systems (e.g., neurological, metabolic, immune, and cardiovascular), which are key regulatory systems of the body [39]. Therefore, it is highly plausible that combined psychosocial and physical environmental exposures may interact to increase or amplify risks of adverse health [1,2,5,7,40,41,42]. By focusing on single pollutant/chemical exposures without consideration of other risk factors acting through common biological systems, health risks may well be underestimated and vulnerable populations may be mischaracterized or overlooked altogether.

## 3. Understanding Biological Mechanisms: Animal Models and Human Biomonitoring

Animal models and human biomonitoring provide insight on the physiological response to chemical and nonchemical stressors, providing a mechanistic basis for epidemiological studies, understanding causation, assessing risk, and developing interventions. Well-designed animal models of multiple exposures can help disentangle the effects of social and environmental stressors, and to elucidate the physiological pathways at play [43]. These models have been used to understand the impacts of prenatal stress, stress exposures during development, or in adulthood or at older ages [44,45,46] and across multiple periods of the life span. Animal models of stress can also provide critical information on the cumulative toxicity of environmental chemicals upon relevant biological pathways. Similarly, measurements of human biomarkers in observational epidemiology, or in controlled exposure studies where feasible and ethical, can elucidate biochemical and physiological pathways by which psychosocial and environmental chemical stressors may interact.

Two STAR CRA projects set out to explore fundamentally different paradigms of interaction between chronic psychosocial stress and chemical exposures. The University of Rochester (Rochester) STAR Project used animal models to test hypotheses in which stress and metals act through common pathways to cause additive or synergistic toxicity. Rutgers University (Rutgers)/Ironbound STAR Project examined children with asthma living in an environmental justice community to examine the hypothesis that chronic psychosocial stress blunts adaptive acute stress responses in asthma, increasing vulnerability to air pollutants. These studies used two different models (animal/human) of interactions that may represent general paradigms applicable to a wide variety of combinations of psychosocial and chemical stressors.

### 3.1. Animal Models to Examine Combined Effects

The Rochester STAR Project examined the cumulative toxicity of metals exposures (i.e., maternal and/or lifetime lead (Pb) exposures, or developmental exposures to methylmercury (MeHg))—in conjunction with stress exposures (i.e., prenatal stress (e.g., prenatal maternal restraint, cold exposure), similar direct stress exposures to the offspring, or the combination of prenatal stress followed by direct stress). The selection of these candidate exposures were based on the known impact of Pb and MeHg on the HPA axis and brain mesocorticolimbic systems, and the fact that these metals and stress lead to similar neurotoxicological consequences [47,48,49,50,51,52]. Moreover, these metals are risk factors that co-occur or even occur successively with psychosocial and physical stressors in many human populations.

**Figure 1:**
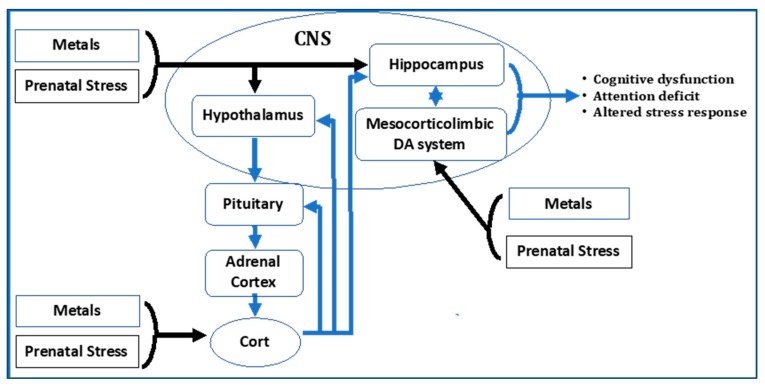
Both metals and prenatal stress act on brain regions including hippocampus, hypothalamus and mesocorticolimbic dopamine regions of brain. Both metals and prenatal stress also impact the HPA axis. This provides common biological substrates for interactions of metals and prenatal stress. HPA axis and brain mesocorticolombic systems have extensive interactions important to mediation of behaviors, including cognition and attention. Modified from Sobolewski et al., 2018 [60].

Findings from the Rochester studies to date indicate that combined exposures to metals and stressors that share biological substrates (here, the HPA axis and brain MESO circuitry) and that produce common adverse effects (e.g., cognitive deficits) can produce enhanced toxicity, or unmask effects of chemical exposures, providing support for the hypothesis of biological interactions consistent with cumulative risk (Figure 1). The enhanced toxicity related to chronic stress is not specific to Pb exposures [47,48,49,51,53,54,55,56,57,58,59], but has been shown for other metals (e.g., MeHg, arsenic (As)) [52,60], suggesting some generalizability. Furthermore, neurotoxicity of developmental exposures to Pb and MeHg can also be enhanced postnatally under conditions of stress to the offspring, for example by early exposure to adversity vs. early positive experience [54].

Importantly, the Rochester STAR Project repeatedly demonstrated that such enhanced neurotoxicity is highly sex-dependent, not with sex-selective effects, but with different profiles of effects by sex and that are persistent across time [47,49,51,52,53,54,59]. Persistent sex-dependent differences in the impacts of Pb and of prenatal stress are also seen on HPA axis function in the 24-h following a dexamethasone challenge test, including sex-dependent enhanced effects of combined lead and stress observed as hypercortisolism in both sexes, but followed by hypocortisolism in males [50]. Such sex differences should be expected, as sex differences actually begin with sex chromosomes, and can involve sex-specific transplacental signals and differences in subsequent epigenetic consequences of stress [61,62,63]. In moving forward, a critical need is for experimental stressors that are better models of human stressors, particularly those related to poverty, that could be applied repeatedly across the lifespan, beginning in gestation, consistent with the cycle of poverty. Common animal stress models such as physical restraint or footshock, for example, may be less relevant to the conditions of poverty than, for example, restricted access to nesting material, which could be related to resource deprivation—albeit human experiences of poverty include critical aspects of social status and psychosocial impacts (e.g., housing insecurity), beyond the direct deprival of physical needs. For that purpose, characterization and validation of resource-deprivation animal models of stress are under development [64].

### 3.2. Human Biomarker Studies to Assess Combined Effects

Biomarkers measured in human subjects can increase understanding of the common biological pathways and mechanisms by which psychosocial stress and air pollution may interact to adversely affect health. Measurement of physiological stress mediators such as cortisol and catecholamines, and their receptors on target or surrogate cells, can quantify responses to acute and chronic psychosocial and chemical stressors. Non-invasive measurements of these intermediate biomarkers in saliva, for example, allows repeated measurements, increasing statistical power in relatively small panel studies that rely on intrasubject temporal variation in exposure to chemical stressors such as air pollution.

Investigators with the Rutgers/Ironbound STAR Project, a partnership with the Ironbound Community Corporation, initiated a panel study designed to test a novel physiological model in which chronic psychosocial stress worsens diesel exhaust-induced asthma exacerbation though biological mechanisms that involve downregulation of adaptive acute stress responses. This hypothesis was based on the premise that the acute stress response mediators, catecholamines and cortisol, are bronchodilatory and anti-inflammatory, respectively, and would counteract asthma exacerbation when incited. If chronic, repeated exposure to a variety of psychosocial stressors causes diminution of this adaptive response, increased susceptibility to agents that trigger asthma exacerbations including air pollutants may result.

Of particular concern among the community members within this seaport-adjacent community were children’s exposures to air emissions from diesel trucks. Previous analyses in this community indicated that heavy truck traffic creates elevated near-roadway concentration of black carbon [65]. In this intensive study, 35 children aged 9 to 14 with mild-to-moderate asthma were monitored for personal exposure to air pollution including black carbon using portable microaethalometers and passive samplers. Over a period of up to 30 days, data on activity, symptoms, medication use, and biomarkers of asthma exacerbation (exhaled nitric oxide and spirometry) were collected by study staff that included trained community workers. Testing the hypothesis required measurement of biomarkers of psychosocial stress, as well as exacerbation of asthma. The intensive, extended periods of personal monitoring of children in the community presented challenges in subject recruitment and retention, which ultimately were met with active engagement of the community partner, the Ironbound Community Corporation. Continuous, 24-h real-time data collection with microaethalometers, set for a 1-minute time base and worn by children, presented data management challenges that included large amounts of data of varying quality that required additional post-processing and imputation of missing data. In preliminary analyses, exposure to previous 24-h mean black carbon concentrations were positively associated with increased exhaled nitric oxide, a marker of airway inflammation, but chronic stress measured by interview did not modify this association [66]. Researchers also measured diurnal variability in salivary cortisol and acute stress responsivity to the Trier Social Stress Test to determine if blunting of acute stress responses is associated with greater sensitivity to diesel air pollution as predicted by their mechanistic model.

## 4. Identifying Environmental Stressors Relevant to Communities

While insights about biological mechanisms are clearly necessary, conducting CRA ultimately requires information on human exposures to chemical and nonchemical stressors. In this context, conventional exposure assessment strategies are often inadequate. Multiple STAR grantees used qualitative research methods that are well-suited to improving the understanding of complex exposure pathways experienced by humans, including the influence of social stressors on health [67]. Qualitative data acquired via individual and focus group interviews contribute to the understanding of people’s behaviors, perceptions of risk, and the social, economic, cultural, and political considerations that influence personal exposure to chemical and nonchemical stressors [67,68,69,70,71,72,73]. Such insights are generally not captured using quantitative methods alone. Qualitative methods are especially important to community-based environmental health research because of their ability to engage residents regarding local environmental health problems.

Multiple studies have used focus group interviews as a preliminary step to help frame or hone research questions, to identify salient exposures in key communities of interest, or to gain a richer understanding of community perceptions, or responses to, environmental hazards [67,74]. In the University of Pittsburgh/West Harlem Environmental Action (WE ACT) STAR Project, for example, spatially-distributed city-wide adult and youth focus groups identified and ranked key neighborhood stressors, which then informed survey development [73]. The qualitative data from the focus groups were analyzed using established methods, and revealed the need to incorporate (and develop robust metrics for) locally-important stressors (e.g., rats and vermin, graffiti, police-community dynamics, gentrification), not commonly included in standard or previously-validated survey instruments or administrative datasets.

In the University of Texas Health Science Center at Houston (UTHealth)/Mano a Mano STAR Project, investigators conducted four focus groups [75] (two among women and two among men) with people of Mexican origin who lived in predominantly Mexican-American neighborhoods in the city of Houston, Harris County, Texas and who were members of the University of Texas MD Anderson Cancer Center Mano a Mano cohort [76]. Focus groups were administered in participants’ preferred language, to identify sources of stress at work, at home, and in their neighborhoods, along with activities and behaviors that may influence exposures to air pollution. Important themes emerged, which were subsequently used to develop closed-ended questions in quantitative questionnaires, including stigma associated with immigration status, and stress associated with neighborhood degradation and safety. Other key sources of stress included their own illnesses, those of family members and neighbors, and economic pressures. In a subsequent study, investigators evaluated the role of stress and air pollution on hypertension among Mexican-origin Hispanics living in Houston [77].

In the Boston University (BU)/Chelsea Collaborative STAR project, one-on-one interviews including both qualitative and quantitative questions were conducted with 354 residents of the City of Chelsea, MA to assess a variety of social and environmental stressors, in addition to defining their neighborhood and drawing a circle around it on a map [78,79]. The research team developed an interview guide that included questions specific to the Chelsea community, was representative and inclusive of the population being studied, while also building on and drawing from existing interview guides, questionnaires and large national surveys (e.g., the Behavioral Risk Factor Surveillance System (BRFSS) and the National Health and Nutrition Examination Survey (NHANES)). Most of the open-ended questions in the BU/Chelsea Collaborative STAR interview guide were created during and after focus groups with community members that identified the community’s specific needs and interests. Responses to the open-ended questions were coded and analyzed to characterize the full range of stressors affecting residents, to explore residents’ meaning or understanding of concerns, and to examine patterns in residents’ responses to questions about their environment. For example, the open-ended questions contain information about specific noises and odors that disturb residents, responses to violence experienced by residents, feelings about safety, satisfaction with local schools, and preferences for use of parks, physical activity patterns, and observations about their residential neighborhoods.

## 5. Challenges in Exposure Assessment for Nonchemical Stressors

While the focus groups and other qualitative methods provide a foundation for exposure characterization of nonchemical stressors, CRA ultimately benefits from quantitative exposure information where viable. Ultimately, community-scale CRA requires either leveraging public data resources in a manner that is relevant to a defined local population or collecting population-specific information in a manner that allows for generalizability to an entire community. Several STAR CRA grantees assessed nonchemical exposures using publicly available national and local scale databases (e.g. NHANES, Census data, city police data) in conjunction with geographic information systems (GIS) and statistical modeling. These approaches were often informed by focus groups and surveys as described earlier, all with a view towards combining nonchemical exposures with chemical exposures.

However, there are many challenges associated with using pre-existing databases and integrating geographic data on chemical and nonchemical stressors which STAR grantees had to address. First, these databases are often collected for very different purposes and the available measures may not accurately or fully capture the specific nonchemical exposure of interest. For example, a researcher may be interested in examining neighborhood housing quality, but available measures may only reflect severe housing violations identified by government authorities. As such, the available measure only partially, and imperfectly, captures the true construct of interest. Additionally, the relevant spatial scale for social processes likely differs substantially (e.g., by neighborhood, within walking distance) from that of chemical exposures (i.e., air pollution can drop sharply with distance from a source). Traffic-related air pollution varies substantially within 50–200 m of roadways [80], whereas social processes may operate at community-, family- or network-level scales [40]. Similarly, just as pollution levels vary temporally (e.g., seasonal pattern in ozone vs. diurnal pattern in traffic-related primary emissions), nonchemical stressors may vary seasonally (e.g., crime rates); as such, there may be temporal misalignment between chemical and nonchemical exposures, as well as with annual or multi-year administrative measures.

Below we highlight approaches taken by STAR CRA grantees to develop exposure assessment for nonchemical stressors and address the challenges of construct validity and scale of aggregation. These studies thus provide important insights, wherein varied types of exposures must each be measured accurately, but also in a manner that can ultimately be combined into a coherent risk assessment framework.

### 5.1. Construct Validity and Publicly-Available Databases for Nonchemical Stressors

Establishing construct validity is challenging for many nonchemical stressors, but particularly for psychosocial stressors. These nonchemical stressors are rarely reflected in aggregate or administrative databases, in part because these constructs rely on individual perception and appraisal [19,81]. In essence, psychosocial stress is not characterized so much by the objective stressor captured in an administrative database (e.g., housing quality, or mean educational attainment), but rather how the individual *feels* about that stressor. Poverty, often used as a proxy for psychosocial stress, is particularly challenging to interpret, because it can reflect (and confound) many aspects of deprivation—some that are primarily material (including housing quality and diet), social (e.g., social support, or access to services), or psychosocial (e.g., job insecurity, hopelessness)—and, indeed, all of these factors are appraised by the individual, and thereby variously contribute to perceived psychosocial stress.

As a result, it is vitally important that researchers validate their use of administrative or other pre-existing measures thoughtfully, to ensure that the measures selected capture the construct of interest, in a manner that is as accurate and unbiased as possible. For physical nonchemical stressors (e.g., neighborhood dilapidation), this could mean on-the-ground surveying of neighborhood conditions, to ensure that the administrative measures accurately capture, or correlate with, the specific aspects of dilapidation that are of interest [82]. For psychosocial stressors, validating the construct requires assessment of individual perception of stressor exposure and/or perceived stress through focus groups, surveys, or other methods to understand stressor appraisal in the community of study. For example, as described previously, the University of Pittsburgh/WE ACT STAR study found, in citywide focus groups, that residents emphasized violence above all other urban stressors [73], and from among 27 administrative stressor indicators, violent crime indicators most strongly correlated with individual survey measures of perceived neighborhood social disorder, perceived stress, anxiety, and depression.

### 5.2. Spatial Scale and Chemical and Nonchemical Exposure Assessment

Stressor data are routinely reported at aggregate scales (e.g., census block), as was used by the UTHealth/Mano a Mano Star Project to estimate exposures to polycyclic aromatic hydrocarbons and diesel particulate matter [83], and administrative boundaries differ by administrative agency or data type (e.g., police precincts, school districts). This resultant spatial misalignment may require researchers to “re-formulate” indicators to a common areal unit for global (overall) analysis, and ideally towards one that approximates lived “neighborhoods.” The BU/Chelsea Collaborative STAR Project asked participants to draw the boundaries of their neighborhood on a map, subsequently transcribed into GIS, and to answer a series of questions about the area they defined. The University of Pittsburgh/We ACT STAR Project compiled data from publicly available sources (e.g., U.S. Census, police data, school district data) and addressed scale of aggregation/spatial misalignment in two ways [84]. First, the researchers asked focus groups and survey respondents to draw their neighborhood. Second, they used proportional areal weighting to reformulate all stressors to that areal unit which best approximated “neighborhoods” (i.e., the United Health Fund (UHF) area) [85]. They validated the method by averaging a known smooth spatial surface (e.g., air pollution maps) for each area (e.g., within each police precinct), then used areal weighing to “re-formulate” the precinct-average concentrations to the UHF. These averages are compared to those derived by directly averaging pollution within each UHF, allowing no more than 5% error [85]. Although computationally intensive, this ‘’validation” is transparent, flexible, and could employ any continuous (smooth) GIS layer (e.g., National Elevation Dataset).

Furthermore, the University of Pittsburgh/We ACT STAR Project needed to develop methods to account for spatial correlation (and thus confounding) across variables, and to account for autocorrelation (clustering) within variables. Shmool et al. used simultaneous autoregressive (SAR) models to assess spatial autocorrelation in bivariate measures of association among stressors. After establishing that spatial clustering did not impact results, conventional exploratory factor analysis (EFA) was used to identify highly-correlated variables, both among and between stressors and pollutants [85].

The issue of spatial scale and misalignment can alternatively be addressed using simulation methods. For example, individual-level data on numerous stressors (i.e., microdata) may be of interest to better understand correlations and clustering among exposures, but these data are often not available with high geographic resolution given privacy concerns. Demographic data may be available at census block resolution but would not provide multi-stressor exposure information. These limitations can be addressed using simulation methods to generate synthetic microdata [86]. In the BU/NorthStar STAR CRA grant, researchers used a simulation modeling technique to characterize spatial patterns of stressors at very fine-scale spatial resolution. First, Levy et al. simulated the sociodemographic characteristics of individuals with census tract resolution in New Bedford, Massachusetts [86]. This involved applying probabilistic reweighting using simulated annealing, which involves selecting a random subset of households from microdata collected as part of the US Census (the American Community Survey) [87] and comparing to aggregate area-level constraints, sequentially replacing individual households to determine whether the model fit improved. The simulated annealing approach helped to ensure that global (overall) rather than local optima were obtained. The researchers then constructed multivariable regression models predicting behaviors and exposures as a function of variables in the synthetic microdata, including for smoking [86], multiple chemical exposures [88], and key public health risk factors of interest to community partners [89]. These models can be applied to determine exposures to chemical and nonchemical stressors at identical spatial resolution, facilitating comparisons and applications in CRAs.

## 6. Statistical Methods for Assessing Combined Effects on Health

Epidemiological investigations of multiple exposures (mixtures) face a daunting challenge. They need to account for a wide array of stressors—chemical stressors as well as any number of nonchemical stressors (e.g., psychosocial stressors, diet, physical activity, noise), each of which may act through different, and often multiple, pathways towards health. Statistical methods, therefore, need be able to account for joint effects of multiple stressors, complex interactions, and non-linear effects. Below, we briefly describe some traditional and novel approaches used by STAR CRA grantees for analyses, including latent variable modeling, structural equation modeling, and alternative approaches for evaluating the health effects of combined exposures.

### 6.1. Latent Variable Modeling

The University of Texas (UT)/Texas City Community Advisory Panel (TCCAP) STAR Project focused on the construction of latent variables, as a means of identifying the predominant stressors and stressor pathways contributing to physiological stress in a community setting. Some pathways included nonchemical stressors reflecting conditions in neighborhoods such as average income, linguistic isolation, perceived crime and neighborhood conditions, low education, poverty and unemployment, and social support. Chemical stressors included air pollutant exposures and air toxics incorporated through a cumulative cancer risk measure compiled by EPA’s National Air Toxics Assessment. UT/TCCAP STAR Project researchers hypothesized that these factors combined and contributed to a measure of cumulative biologic impact (akin to McEwen’s concept of allostatic load) [90]. To evaluate the association with these stressors, acting together or alone, on health, researchers used latent variable modeling. The data were drawn from a Texas City community survey, blood assays, air pollution CMAQ (Community Multiscale Air Quality Modeling System) models (www.epa.gov/cmaq/cmaq-data), and the US Census, and included both individual and neighborhood-level variables. To create the latent factors, UT/TCCAP STAR Project researchers first used a covariance decomposition procedure similar to confirmatory factor analysis, combining the stressor variables into empirical groupings [91]. The final model identified three latent factors: (1) ‘place-based stressors’ comprised of census block and census-tract-level linguistic isolation, unemployment and low education indicators; (2) individual-level ‘disadvantage’ comprised of individual education, household income and race and ethnicity; and (3) individual-level measures related to ‘psychosocial stress’, comprised of individual scores on stress, an assessment of coping and total social support scales. For example, a one-standard deviation increase in the factor related to air toxics-related area cancer risk was associated with a 0.05-standard deviation increase in the composite biologic score, possibly mediated via the latent factors of ‘place-based stressors’ and individual ‘disadvantage’.

### 6.2. Structural Equation Modeling (SEM)

Multiple STAR grantees applied structural equation modeling (SEM) to address high-dimensional data and complex interactions. SEM is particularly well-suited for cumulative risk assessment, as it combines linear regression, path analysis, and factor analysis, allowing for the joint effects of multiple stressors to be formally evaluated [92]. Specifically, it allows for simultaneous models of predictors of exposure and predictors of health outcomes, with consideration of whether sociodemographic variables are associated with health outcomes directly or through environmental exposures. In the BU/NorthStar STAR grant, researchers successfully applied SEM to data from NHANES, simultaneously modeling predictors of blood pressure and predictors of multiple environmental exposures using candidate variables available from public data resources [93]. For example, researchers found that systolic blood pressure was associated with polychlorinated biphenyl (PCB) exposures as well as multiple sociodemographic covariates (e.g., age, gender, race/ethnicity) and factors such as body mass index (BMI) and smoking status. The SEMs revealed some of the complex pathways between exposures and outcomes that must be considered within cumulative risk assessment (i.e., smoking was associated with higher Pb and Cd exposures but lower BMI and PCB exposures, and also had a direct inverse association with systolic blood pressure). In addition, SEM was used to develop models of multiple chemical exposures as a function of publicly available covariates [88] However, SEM requires a large sample size to yield robust findings and has challenges with non-normal and categorical data [88]. Although some of these challenges can be reasonably addressed (i.e., by log-transforming data and developing indices from multiple categorical variables) and are common to multiple statistical techniques, SEM is not suitable for all applications. In a parallel epidemiological analysis for the BU/NorthStar STAR grant leveraging exposure and health outcome data from a long-standing birth cohort study, SEM did not yield interpretable findings. This was attributed to the sample size and the relatively modest and non-linear effect of individual chemical stressors on ADHD-like behavior as shown previously [94].

### 6.3. Alternative Approaches for Evaluating Complex Interactions

As part of the UTHealth/Mano a Mano STAR Project, investigators developed an analytic approach for evaluating interactions between independent variables, which are categorized using a specific quantile breakdown for continuous variables. The advantage of this method is that it simplifies interpretation of interaction terms in logistic regression. In this approach, specific categories of a chemical and nonchemical stressor define the rows and columns of a matrix of cells, similar to what is presented in a multi-way analysis of variance (ANOVA) model. In this matrix, the overall probability distribution of the outcome, as well as the mean of the outcome probabilities (i.e., the proportions of individuals with the outcome of interest) of each cell are tabulated. After taking the logit transformation of these probabilities, an ANOVA model can be applied to test interactions between specific values of the chemical and nonchemical stressors, aided by a bootstrapping technique to estimate the variance of each cell. Specifically, the null hypothesis for testing the interaction between a chemical and nonchemical stressor can be expressed as the logit (cell probability) – logit (corresponding column marginal mean probability) – logit (corresponding row marginal mean probability) + logit (overall mean probability). Note that taking the logit transformation of these probabilities helps make the observed cell probabilities approximately normally distributed. Additionally, this method can be modified for testing higher-order interactions effects.

The researchers also applied a method using a statistical isobole to assess interactions. The statistical isobole draws upon the framework of a biological isobole often used in pharmacological research for identifying interaction effects of two drugs (see, for example, Tallarida (2012)) [95]. The statistical isobole allows analysts to classify each pair of values (of the chemical and nonchemical stressor) as representing a synergistic, antagonistic or no-interaction effect. This approach also derived statistical tools to make inferences on collected data about whether interaction is present. The results suggest that proposed method may be more robust than traditional methods because it allows for making inferences about interactions beyond the form of a product term.

The BU/Chelsea Collaborative STAR investigators developed an Open Source software for examining the hierarchical structure of data sets. The software, Epi-ACE (v. 07-15-2014, available at sourceforge.net) enables analysts to scale attributes, import data and draw lattice diagrams using Formal Concept Analysis (FCA) [96]. FCA is a mathematical approach for analyzing relationships in datasets. The team identified two major functions of direct relevance to CRA. One is to allow creating a lattice representation of data that illustrates hierarchical relationships (dependent and independent variables, exposures and outcomes), clustering and co-occurrence of stressors and outcomes. Perhaps similar to a scatter plot generated with regression analyses, the lattice illustrates relationships between dots on the plot, in a similar manner as SEM or path analyses. Each union or dot includes multiple stressors. There is no x- or y-axis, as the lattice is a complete structure. The other function is the ability to quantify the relationships among these clusters to generate prevalence odds ratios and confidence intervals for specific exposure, outcome and covariate combinations.

## 7. Discussion

Understanding the totality of human exposures, and how environmental exposures to multiple chemicals and chemical mixtures affect disease pathogenesis, are longstanding critical problems facing public health [13,97]. Federal environmental policy makers have been grappling with this challenge since at least the late 1980s when concerns emerged about potential environmental and health effects from multiple chemical exposures associated with abandoned and uncontrolled hazardous waste sites [98]. Despite the growing evidence of cumulative health effects of multiple chemical exposures, including evidence about additive and synergistic effects of joint exposures to chemicals and social stressors [2,6,59,93,99,100], little progress has been made to routinely apply CRA to environmental regulatory and policy decision-making. Specific chemical mixtures have been analyzed for some time, in part because of statutory language (i.e., in the Food Quality Protection Act, which required examination of cumulative risks of pesticides with common mechanisms of toxicity), but policy analyses have not generally considered broader categories of mixtures that include nonchemical stressors. Policy-makers have reasoned that this is due to a general scarcity of appropriate data on chemical mixtures, the paucity of toxicological data on the biological mechanisms of adverse health effects of mixtures and multi-stressor exposures, and a shortage of verified analytical frameworks for cumulative risk. However, the EPA STAR CRA grantees have demonstrated that many of the necessary analytical methods to support CRA are readily available. They involve a variety of techniques, existing and novel, including both quantitative and qualitative approaches, reinforcing that there is no one ideal approach for CRA given the wide range of applications and settings. While the STAR grantees used diverse approaches to understand the combined impacts of chemical and nonchemical stressors, each of the studies detailed above made important contributions to furthering methods for CRA.

The projects developed and tested methods for some of the building blocks of CRA. Each project implicitly or explicitly addressed some aspect of planning, scoping and problem formulation, although this was not the primary focus of any individual project. Examples of methods and tools relevant to CRA planning and scoping employed by the grantees include focus groups and interviews among community residents to identify locally-relevant stressors, which were subsequently incorporated into survey instruments. STAR CRA projects found that asking open-ended questions via these qualitative methods was particularly helpful in revealing or identifying important psychosocial stressors in residents’ lives that are not usually captured by existing data sources (e.g., unstable employment, language barriers, and immigration status). Many of these CRA STAR grantees applied community-engaged research approaches as a strategy to integrate communities’ knowledge into research on environmental exposures, as well as to build community capacity to address environmental health problems. However, these qualitative and community-engaged approaches are most relevant to local-scale assessments and may not be applicable to national-scale assessments, which are generally needed for regulatory environmental decision making by EPA.

Elucidating how chemical and nonchemical stressors may act via the same biological systems/substrates can help provide focus for CRAs, given the numerous candidate stressors. Laboratory/experimental studies have historically provided the basis for risk assessment. Increasing toxicological investigations that not only examine chemical mixtures but also animal models of combined chemical and nonchemical stressor exposures will be valuable in validating and identifying possible biological pathways and informing the epidemiological studies as illustrated by one of the CRA grantee projects. However, many methodological challenges remain for integrating insights from both toxicological and epidemiological studies for assessing combined effects of multiple stressors. First, there are many available animal stress models, so selecting the right model requires careful thought [44]. The animal stress models should be selected based on the hypothesis to be tested and closely simulate, to the extent possible, the biological equivalent of chronic psychosocial stress in human populations [45] (e.g., the human experience of poverty, discrimination or stigma). Animal models more akin to social stressors in human include maternal separation, isolation housing, intruder entry into the cage, and odors of predators [46]. However, many animal models for psychological stress include physiological stress components (e.g., forced swim [101,102], cold exposure [46], foot shock) to which animals often show adaptation as these stressors become controllable and predictable. Animal models incorporating multiple varied stressors [103,104,105] may better simulate the complexity and unpredictability of many impoverished human environments.

Further interactions among many chemical and nonchemical exposures likely have non-linear effects on health (dose-response curves), just as many individual chemical exposures have non-linear effects. Some exposures may exert notable biological effects *only* above a given dose (i.e., a threshold effect), while others may exert a constant, non-varying biologic effect above a given dose (i.e., a saturation effect) [40]. For example, one earlier study of indoor environmental interventions to reduce childhood asthma symptoms in Boston revealed that, for families reporting substantial fear of community violence, there were no observable benefits of indoor environmental interventions; this result suggested that the impact of this strong urban stressor may, at very high exposures, act to nullify any benefits of physical environmental improvements [104]. In contrast, at lower doses, urban violence has been associated with heightened impacts of physical environmental exposures [105]. Because of these complex dose-response effects—which are especially challenging for interactions among multiple exposures which may have independent non-linear effects—there is a great need for more sophisticated statistical methods to address mixtures, and for more toxicological research elucidating the biological pathways and dose-response curves for combined effects on respiratory [106] and other disease outcomes. Ultimately, there needs be richer incorporation of toxicological information into epidemiologic studies of these interactions, and vice-versa, to provide the critical knowledge base for CRAs.

The STAR grantees provided significant insights related to both exposure assessment and dose-response modeling, which are important for CRA. For exposure assessment, grantees confronted the challenge of characterizing exposures to key nonchemical stressors. In the absence of direct measurements of psychosocial stress, grantees used a range of publicly-available datasets on demographics, health characteristics, and other geospatial information that may be significantly associated with psychosocial stress. However, as some grantees noted, limitations of the interpretability of administrative data include potential differential misclassification, issues of validity of the social stressor construct of interest, and spatial and temporal units of aggregation. Issues of geospatial accuracy and resolution were addressed through multiple novel statistical techniques, while some grantees directly validated that the administrative data truly represented psychosocial stress. Broadly, given that exposure assessment has often been an underappreciated aspect of CRA, the advancements made across the range of EPA STAR grants were helpful in moving the field forward.

For dose-response modeling, researchers used a variety of advanced statistical techniques to better elucidate the combined effects of multiple stressors, a challenge that extends well beyond the context of CRA [107]. Structural equation modeling was a promising technique utilized by multiple grantees, but given limitations in sample size, data structures, and interpretation of outputs, it may only be applicable to a subset of investigations. Similarly, techniques such as statistical isoboles or lattice diagrams can provide considerable insight, but some of these strategies would be challenging to apply directly to a quantitative CRA. More generally, what these collective investigations reinforced is that epidemiological investigations conducted with a CRA application in mind could provide more relevant information than available from post hoc syntheses of the published literature.

Although the STAR grantees collectively addressed a number of the building blocks of CRA, some significant gaps remain. First, CRA is ultimately most valuable when it is decision-relevant; in the absence of a specific risk management or policy decision that is calling for insight from CRA, it is difficult to narrow the scope of stressors under consideration [13]. While linkages with community partners ensured that the seven STAR CRA projects were relevant to local concerns, their specific insights are challenging to implement in a decision-making context. In addition, many of the methods utilized are predicated on the availability of locally-relevant exposure information and health databases, which would limit broad-based application. Rarely will EPA or others conducting CRAs for policy decisions have the opportunity to conduct new epidemiological investigations or to collect new exposure information. Ultimately, CRAs will only be successful if exposure scientists, epidemiologists, toxicologists, and others develop models and databases with a CRA end use in mind. For example, there is a range of potential policy applications of CRA, which may employ methods described herein, including identifying vulnerable overburden communities for technical assistance, enhanced monitoring or enforcement of environmental laws and regulations; targeting neighborhoods for financial investments to reduce pollution and build community resilience; informing multi-pollutant control strategies and systems approaches to reduce chemical and nonchemical exposure burdens; and ultimately informing the setting of health-based standards for chemical pollutants and permitting decisions.

## 8. Conclusions

In spite of the gaps described above, these seven projects collectively provided three key advances. First, they demonstrated approaches for community engagement in CRA. As articulated elsewhere [17], there were variable strategies for community engagement and numerous lessons learned, but there was a collective recognition of its importance for planning, scoping, and problem formulation, as well as for data analysis. Second, they demonstrated the ability to characterize exposures to psychosocial stress, or, in the absence of this information, to characterize exposures to multiple stressors that may act as surrogates for, or predictors of, psychosocial stress. Finally, they demonstrated through both toxicological and epidemiological investigations that the health effects associated with chemical stressors are modified in important ways by exposure to psychosocial stress and other nonchemical stressors.

## Figures and Tables

**Table 1:** Overview of the Science to Achieve Results (STAR) understanding the role of nonchemical stressors and developing analytic methods for cumulative risk assessments grants.

Short Title	Full Title	Institution(s)	Link to Grant Abstract and Annual Reports	Exposures		Outcomes
				Psychosocial/Nonchemical Stressors Evaluated	Pollutant/Chemical Stressors Evaluated	Health Outcomes Evaluated
The BU/Chelsea STAR Project	New Methods for Analysis of Cumulative Risk in Urban Populations	Boston University; The Chelsea Collaborative	https://cfpub.epa.gov/ncer_abstracts/index.cfm/fuseaction/display.abstractDetail/abstract/9278/report/0	Crisis In Family Systems (CRISYS) survey items: financial, legal and home issue domains; Neighborhood/Block Conditions, IV Environmental Assessments, Exposure to Violence Assessment, Inner City Asthma Study, The Multigroup Ethnic Identity Measure, Reactions to Race; food accessibility, use of parks, perceptions of noise and odor.	High traffic roadways, toxic release inventories, land uses.	Self-rated health; diagnosed diabetes, heart attack, heart disease, asthma, emphysema/respiratory disease, overweight, arthritis, hypertension, psoriasis, vitiligo, cancer, depression/mental health and chronic disease; symptoms
The BU/NorthStar STAR Project	Effects-Based Cumulative Risk Assessment in a Low-Income Urban Community near a Superfund Site	Boston University; NorthStar Learning Centers	https://cfpub.epa.gov/ncer_abstracts/index.cfm/fuseaction/display.abstractDetail/abstract/9144/report/0	Sociodemographic proxies (education, race/ethnicity, income), maternal smoking during pregnancy, maternal stress during pregnancy (characterized in part by community violence), diet, access to health care.	ADHD-like behavior: Blood Pb (cord blood and at various ages), serum polychlorinated biphenyls (PCBs), serum DDE, hair mercury, ETS exposure approximated by questionnaire data and modelsBlood pressure: Blood Pb, blood Hg, blood Cd, ETS	ADHD-like behavior, blood pressure
The University of Pittsburgh/WE ACT STAR Project	Community Stressors and Susceptibility to Air Pollution in Urban Asthma	University of Pittsburgh; West Harlem Environmental Action (WE ACT)	https://cfpub.epa.gov/ncer_abstracts/index.cfm/fuseaction/display.abstractDetail/abstract/9279/report/0	*Community stressors* were identified in citywide focus groups. GIS indicators were collected from administrative data (yrs 2008–2010) on crime and violence, mental and general health, built environment, healthcare access, noise disruption, child-specific stressors (e.g., schools conditions), socio-economic position. Citywide surveys measured perceived stress, anxiety, depression, caregiver stress, urban life events, unfair treatment, social support, and perceived neighborhood disorder, social capital, and violence.	DOHMH New York City Community Air Survey (NYCCAS) city-wide pollution (PM_2.5_, NO_2_, EC, SO_2_, O_3_), 2008–2010. Environmental Protection Agency (EPA) AQS regulatory daily_._	Child asthma exacerbation and morbidity
The Rutgers/Ironbound STAR Project	Effects of Stress and Traffic Pollutants on Childhood Asthma in an Urban Community	Rutgers, The State University of New Jersey; Ironbound Community Corporation (ICC)	https://cfpub.epa.gov/ncer_abstracts/index.cfm/fuseaction/display.abstractDetail/abstract/9277/report/0	UCLA interview, focus groups, stress reactivity, glucocorticoid and b2-adrenergic receptor levels.	Personal real-time black carbon, 24-h NO_2_ by passive sampler, EPA central site priority pollutant	Emergency department data on asthma exacerbation measured as symptoms, medication use, exhaled nitric oxide, spirometry
The Rochester STAR Project	Combined Effects of Metals and Stress on Central Nervous System Function	University of Rochester School of Medicine and Dentistry	https://cfpub.epa.gov/ncer_abstracts/index.cfm/fuseaction/display.abstractDetail/abstract/9275/report/0	In animal models, the stressors are directly imposed and measures of its efficacy can include any changes in function of the hypothalamic-pituitary-adrenal (HPA) axis or changes in behavior or brain function.	Blood lead and brain measures of metals by atomic absorption spectrometry	Cognitive function, IQ, attention deficit
The UT/TCCAP STAR Project	Analytical Strategies for Assessing Cumulative Effects of Chemical and Nonchemical Stressors	University of Texas; Texas City Community Advisory Panel (TCCAP)	https://cfpub.epa.gov/ncer_abstracts/index.cfm/fuseaction/display.abstractDetail/abstract/9276/report/0	Neighborhood stressors (crime, infrastructure, income distribution, segregation) Social stressors (poverty, employment, discrimination, access to care, educational attainment) Psychosocial stress (perceived stress, perception of environmental risk, self-reported coping, social support.	Chronic: EPA National Air Toxics Assessment 2005 ambient concentrations of air toxics (cumulative cancer risk). Acute: Upset event releases of toxics, monitoring data, ozone warning days, PM alerts, high allergen counts, self-reported exposures.	Allostatic load, SR-36, glucose and lipid markers, antibody titers, inflammatory markers, current conditions, current meds
The UTHealth/Mano a Mano STAR Project	Hypertension in Mexican-Americans: Assessing Disparities in Air Pollutant Risks	University of Texas Health Science Center at Houston (UTHealth) School of Public Health; MD Anderson Cancer Center; National Chiao-Tung University	https://cfpub.epa.gov/ncer_abstracts/index.cfm/fuseaction/display.abstractDetail/abstract/9147/report/0	Individual-, family- and neighborhood-level stressors identified through community focus groups:—Individual level (anxiety/depression, aches and pains, trouble sleeping, worrying about health/money/time for oneself)—Family level (family illness/separation, domestic violence, trouble with children)—Neighborhood level (noise, traffic, litter, safety, violence at children‘s school)—Other social stressors (coming into contact with authorities, feeling discriminated against due to race/ethnicity or immigration status)—Stressors at work (worrying about working too hard or harmful substance exposures)	Publicly available data sets from the Texas Commission on Environmental Quality (TCEQ); concentrations of particulate matter ≤2.5 micrometers in aerodynamic diameter (PM_2.5_) and ozone (O_3_).	Self-reported hypertension
